# The impact of the COVID-19 pandemic on health outcomes in delusional disorder: A systematic review

**DOI:** 10.1192/j.eurpsy.2023.1669

**Published:** 2023-07-19

**Authors:** E. Román, M. Natividad, M. V. Seeman, E. Izquierdo, E. Martínez, E. Rial, A. Alvarez, A. Guàrdia, J. A. Monreal, A. González-Rodríguez

**Affiliations:** 1 Mental Health, Mutua Terrassa University Hospital. University of Barcelona (UB). CIBERSAM; 2 Mental Health, Mutua Terrassa University Hospital. University of Barcelona (UB). Fundació Docència i Recerca Mutua Terrassa, Terrassa, Spain; 3 Psychiatry, University of Toronto, Toronto, Canada; 4 Mental Health, Mutua Terrassa University Hospital. University of Barcelona (UB). CIBERSAM. Fundació Docència i Recerca Mutua Terrassa; 5 Mental Health, Mutua Terrassa University Hospital. Univerity of Barcelona (UB). CIBERSAM. Fundació Docència i Recerca Mutua Terrassa. Inst. Neurociències. UAB, Terrassa, Spain

## Abstract

**Introduction:**

The health impact of the COVID-19 pandemic has been widely recognized in both physical and mental health. Relatively little attention has been paid to patients with delusional disorder (DD).

**Objectives:**

Our goal was to synthesize the known mental and physical health consequences of the COVID-19 pandemic in patients diagnosed with DD.

**Methods:**

A systematic review was carried out using the PubMed and Scopus database (2019-October 2022) following the Preferred Reporting Items for Systematic Reviews and Meta-Analyses (PRISMA) statement. Search terms: “delusional disorder” or “delusional disorder” AND “COVID-19 OR SARS-CoV2.” Inclusion criteria: 1)DD according to DSM/ICD, 2)languages: English, French, German and Spanish, 3)studies reporting health consequences of COVID-19 pandemic. From a total of 615 records, 6 were included: meta-analysis (n=1), cross-sectional studies (n=2), retrospective study (n=1), case reports (n=2).

**Results:**

A full third of patients with psychosis (including DD) presented with increased psychiatric symptom severity, reportedly activated by increased daily life stress. Suicidal behavior was reported in a previously undiagnosed DD patient in association with a worsening clinical picture. Perhaps surprisingly, admissions for DD in 2020 were lower than in 2019. The duration of hospitalization was, however, longer. There was a report of new onset DD with delusional material centred on COVID. There was also a report of COVID-19 symptoms being more severe in DD patients than in the larger community.

**Conclusions:**

Health emergencies affect the seriously mentally ill more than other community members. Awareness and outreach can help to maintain treatment adherence and minimize risk of psychotic exacerbation.

**Disclosure of Interest:**

None Declared

